# Job Burnout, Work-Family Conflict and Project Performance for Construction Professionals: The Moderating Role of Organizational Support

**DOI:** 10.3390/ijerph15122869

**Published:** 2018-12-14

**Authors:** Guangdong Wu, Yue Wu, Hongyang Li, Chenglong Dan

**Affiliations:** 1 Department of Construction Management, Jiangxi University of Finance & Economics, Nanchang 330013, China; wuguangdong@jxufe.edu.cn (G.W.); obwy1994@163.com (Y.W.); 2 School of Civil Engineering and Transportation, South China University of Technology, Guangzhou 510641, China; cthyli@scut.edu.cn

**Keywords:** construction professional, organizational support, work-family conflict, job burnout, project performance

## Abstract

Construction professionals are prone to work-family conflict and job burnout, which in turn can affect project performance during implementation of delivery. To cope with this, a questionnaire survey was undertaken with construction professionals in the Chinese construction industry and 373 valid responses were received. A theoretical model introducing organizational support as a moderating variable was developed and tested with structural equation modeling. The results showed that there is a positive correlation between work-family conflict and job burnout for construction professionals. Involving organizational support can alleviate the impact of work-family conflict on professionals’ job burnout. Meanwhile, both work-family conflict and job burnout have negative effects on project performance. Therefore, an atmosphere and culture of humanized management should be established within the construction enterprises. Additionally, organizational incentives such as regulations and rules should be formulated assisting employees achieve work-family balance. Such incentives could contribute to the construction industry and improve project performance. Furthermore, this study provides a new theoretical perspective for the management of job burnout and work-family conflict in the construction industry, complementing the existing body of knowledge.

## 1. Introduction

Job burnout has become a serious problem in the construction industry, which is a typical task-driven industry [1,2]. Construction has always been regarded as an industry with high levels of work pressure and stress [3,4]. In order to achieve optimal project performance, construction professionals are often expected to bear great pressure during the whole project life cycle. This puts construction professionals at high risk of experiencing job burnout [5]. Over the past decades, construction projects have tended to be large-scale, complex and integrated [6]. These characteristics of construction projects frequently require professionals to devote more time and energy to work, which could lead to job burnout. Therefore, job burnout inevitably occurs in the construction implementations for construction professionals [7]. Job burnout will not only affect a professional’s personal daily life, but will also lead to time and cost overruns, and reduce customer satisfaction. The overall result could be a negative impact on project performance [8,9,10].

As a labor-intensive industry, the construction industry has high-risk and discrete nature, with heavy workloads and long construction periods. The use of mobile devices enables construction professionals to connect to work regardless of time or location, effectively transcending the boundaries of work and family [11]. These factors all combine to cause construction professionals to work long hours under immense work pressure, and may limit the ability of them to effectively perform both their work and family roles [12]. Long hours of work are associated with dissatisfaction in the quality of familial and marital relationships [13]. Construction professionals experience heavy workloads and challenging tasks may go home with bad emotion and show a different demeanor with family members [14]. As such, construction professionals suffer from serious work-family conflict [15]. Work-family conflict has negative consequences for professionals’ work and family life, physical and mental health and well-being [16,17,18,19]. This type of conflict increases professionals’ work stress and triggers job burnout [20,21,22]. Furthermore, increased job requirements may limit the ability of employees to effectively participate in their work and family roles. Thus, work-family conflict may promote job burnout for construction professionals [12].

The increased work-family conflict being experienced by construction professionals also leads to more severe job burnout [23], which in turn results in poorer project performance [24]. If the supervisors could give more organizational support to their employees, the enthusiasm of the staff could be motivated, and thus, project performance might also be improved [25]. Most of the existing studies related to job burnout focus on the relationship between job burnout and job satisfaction [26,27,28]. However, the critical factors affecting employees’ burnout, such as work-family conflict and organizational support, as well as the project performance problems caused by job burnout—are ignored to some extent. In order to address such research gap, this study introduces organizational support as a moderating variable to construct a theoretical model to investigate the relationship between job burnout, work-family conflict and project performance for construction professionals in China. An empirical test is also conducted adopting structural equation modeling. The objectives of this study are as follows: (i) to explore the relationship between work-family conflict and job burnout; (ii) to determine whether a significant correlation exists between job burnout and project performance; (iii) to verify whether work-family conflict is significantly related to project performance, and (iv) to confirm that organizational support plays a moderating role between work-family conflict and job burnout. Few studies have explored the relationship between job burnout, work-family conflict, project performance and organizational support in the construction industry. Therefore, this study can contribute to a theoretical and practical insight into the management of work-family conflict and job burnout, thereby providing an effective reference for construction enterprises to improve project performance.

## 2. Theoretical Background

### 2.1. Job Burnout

The term “job burnout” was first proposed by Freudenberge [29], and was used as a reference to occupational psychological syndrome. Subsequently, Maslach [30] carried out a further study on this issue focusing on people in the service industry. He proposed that job burnout can be measured from three dimensions: emotional exhaustion, cynicism and low professional efficacy. In recent years, studies relating to this issue have all been based on Maslach’s theory. Many theoretical models and mechanisms have been put forward in the past to systematically explore the causes and consequences of job burnout. Efforts have also been made to further improve measurement accuracy and applicability [31,32]. Of the existing models, the most widely used forms in various research fields include: the job demand-control model [33], the job demand-control-support model [34], the effort-reward imbalance model [35] and the job demand-resource model [36]. Based on these models, researchers have conducted statistical analyses, correlation analyses or structural equation modeling of burnout. However, few researchers have focused their attention to this issue specifically in terms of the construction industry [10]. Leung et al. [8] conducted research that examined psychological stress in construction professionals and suggested that support from workmates is the most direct way to prevent construction professionals from burnout. However, Leung’s research perspective not only focuses on burnout, but also measures long-term psychological stress in conjunction with two other stress indicators (e.g., work stress and physiological stress) to verify their impact on project manager performance. Yang et al. [2] analyzed the antecedent variables of the job burnout of construction project managers from the perspective of organizational justice, based on a job demand-resource model. They proposed that material support from the project organization can reduce the level of employee burnout more effectively than can spiritual support. Lingard et al. [23,25,37,38,39,40,41] conducted many studies on work-family relationship of construction professionals. These studies have made outstanding contributions in revealing the work stress problem, the related mechanism of construction professionals, and in providing effective stress mitigation measures. Lingard [37] and Yip et al. [42] used the Maslach Burnout Inventory-General Survey (MBI-GS) scale in Australia and Hong Kong to investigate job burnout in engineers working for consulting companies and construction companies. Both studies confirmed that construction professionals have high levels of job burnout and proposed that organizational support such as regular holidays is a good way to prevent employees from burnout. 

The job burnout of professionals in the construction industry is due to the dual pressures of task goal and communication/coordination. Therefore, the definition of job burnout should be different from those for general professionals. In addition to following Maslach [30] with regard to the definition of job burnout of general employees (emotional exhaustion and low sense of achievement), this study adjusts the definition according to a cynical perspective - expanding the definition of job burnout to include indifference or alienation towards work and colleagues. Thus, the factors of (i) whether the degree of interest in the job decreases, and (ii) whether the degree of enthusiasm for the work and colleagues’ decreases are also introduced into this study.

### 2.2. Work-Family Conflict

Kahn [43] proposed that work-family conflict is a form of incompatibility between work and family in some areas. Greenhausand et al. [44] divided work-family conflict into time-based conflict, strain-based conflict and behavior-based conflict. Work-family conflict includes both work interference with family and family interference with work (FIW) [45]. In the past decades, increasing attentions are being paid to work-family conflict in the construction industry. Liu et al. [46] investigated work-family conflict from the perspective of time-based conflict, strain-based conflict and behavior-based conflict among Chinese project managers. Bowen et al. [47] explored the relationship between work contact, work-family conflict and the health issues such as psychological distress and sleep problems among South African construction professionals and found that empowering staff with greater job control will help them to alleviate the work-family conflict and its bad consequences. Ng et al. [48] proposed that work-family conflict is one of the most difficult stress factors for construction professionals in the construction industry. Lingard et al. [37] found that work-family conflict of construction employees can be predicted by excessive work demands. Francis et al. [49] compared the work-family conflict experience of public and private sector construction workers and proposed that flexibility in managing work and family obligations is helpful in reducing work-family conflict. Xia et al. [14] used project commitment as a mediator to analyze the relationship between work-family conflict and project citizenship behavior and suggested that family-friendly measures should be adopted to decrease project managers’ work-family conflict. Other researches in the construction industry indicated that competitive bidding [50] and strict project planning [51] lead to longer working hours, which in turn has a significant impact on employee work-life pressure. Working hours, supervisors’ support and job flexibility may affect the degree of work-family conflict [52]. Wu et al. [15] found that increased job flexibility and organizational support have positive effects on reducing work-family conflict and on improving the performance for construction professionals. Work-family conflict acts as a link between work plans and employee burnout [39], while organizational support and other forms of job support can alleviate work-family conflict and burnout [25]. Turner et al. [52] pointed out that the key factors that affect work-family conflict based on time and strain for construction professionals were the length of working hours, the rationality of working hours, and the flexibility of management. Based on these findings, he proposed that work-life programs are good strategies to reduce the work-family conflict. Additionally, in the case of long working hours, greater flexibility in the working hours of construction professionals helps them alleviate their work-family conflict [53,54].

In the construction industry, work-family conflict can be defined as the incompatibility of role pressures between work and family for construction professionals. In other words, work-family conflict is a form of conflict between work and family roles [16,44]. Therefore, the measurement of work-family conflict can be divided into two aspects: work-family conflict and family-work conflict. The former refers to the intrusion of and interference of work with the family, while the latter to the intrusion of and interference of the family with the work.

### 2.3. Organizational Support

Eisenberger et al. [55] defined organizational support as the overall perception and belief of employees about how an organization views their contributions and cares about their interests. McMillin [56] divided organizational support into instrumental support and social emotional support. Allen et al. [57] proposed a three-dimensional organizational support integration model, in which organizational support is divided into instrumental support, emotional support, and superior support. Studies have confirmed that organizational support is an effective way to reduce job stress and job burnout in construction professionals [58,59]. Leung et al. [59] found that informal organization support is more effective than formal organizational support in addressing the stress of construction estimation professionals. Lingard et al. [25] revealed that support from companies, supervisors and colleagues has different effects on the job burnout. Yang et al. [10] pointed out that Chinese project managers may be at high risk of experiencing job burnout due to long-term job stress, if they do not have enough organizational support. Turner et al. [52] proposed that organizational support is one of the most significant factors affecting work-family conflict for construction professionals. Anderson et al. [60] argued that organizational support can reduce the adverse consequences of work-family conflict.

Therefore, the organizational support of the construction industry can be defined as the attitude of the construction company toward employee’s contributions, as well as the concern of the construction company for the welfare of its employees [55]. Referring to the questionnaire design used in Yang et al. [2] and Hao et al. [61], this study measures organizational support from three dimensions i.e., support of work, identification of value and concern for employees’ interests. 

### 2.4. Project Performance

Project performance is a key indicator for evaluating the success of a construction project [62,63]. Traditional project performance is narrowly defined as an iron triangle of cost, time and quality. It is easy to see the iron triangle that leads to the short-term optimal and long-term sub-optimal consequences of project performance [64,65,66]. With the development of the project environment, early project performance criteria may not suitable for the current or later stages of the project [67]. Customer satisfaction and other relevant stakeholder (a group or organization that has interest or concern in a project, such as the owner, the contractor, the sub-contractor, the designer, the consultant, the supervisor and so on) satisfaction criteria should also be taken into account when evaluating project performance [68,69,70]. Zheng et al. [71] suggested that implementation of the owner’s strategic goals, end-user satisfaction and other stakeholders’ satisfaction should also be included when evaluating project performance. Kim et al. [72] proposed that the main evaluation indicator of project performance is customer satisfaction.. Joslin et al. [73] used project efficiency, organizational effectiveness, project impact, stakeholder satisfaction and future potential collaboration as project performance evaluation indicators. Luo et al. [6] stated that project performance indicators include time, cost, quality, employee health and safety, environmental performance, participant satisfaction, customer satisfaction and business value. Some scholars even assessed project performance from the whole life cycle, including the project delivery phase and post-delivery phase [74,75,76,77]. These scholars mainly examined project cost, duration, and quality during the delivery phase, and they focused on customer and project participant satisfaction evaluation, customer impact evaluation, and the evaluation of business value during the post-delivery phase.

In the context of the construction industry, the interests of various stakeholders are different. Thus, the definition of project performance varies from one stakeholder to another [78,79]. The main objective of this study is to explore the effects of job burnout and work-family conflict on the project performance. Therefore, the inherent nature of construction projects is combined with the characteristics of employees working in construction enterprises. This study evaluates project performance in terms of the overall project objectives and stakeholder satisfaction (i.e., in terms of cost, duration, quality, and partner satisfaction with the project completion process) and the various participants’ willingness to continue to cooperate in future projects.

## 3. Hypotheses Development and Theoretical Model

### 3.1. Hypotheses Development

#### 3.1.1. Work-Family Conflict and Job Burnout

As a source of stress, work-family conflict brings negative effects, e.g., low efficiency levels, becoming demoralized, job burnout, absence from work and high employee turnover rates [44]. Based on the conservation of resources (COR) theory, people strive to acquire and maintain resources that help achieve goals [80]. Resources include conditions, personal characteristics, objects, and energies. Employees feel unable to fulfill work and family responsibilities may lead to potential or actual loss of resources like divorce and being fired at work when they experience work-family conflict [14]. Stress occurs as a result of resources loss in the process of balancing work and family roles [81]. The threat of potential or actual loss of resources is a major factor in the stress process and can even lead to job burnout [82]. Then, they may take actions to minimize resource losses and protect remaining resources to address stresses caused by work-family conflict [81]. For construction professionals, due to the pressure caused by excess working hours, changing needs and complex tasks [83], increasing job requirements may limit the ability of construction professionals to effectively perform both their work and family roles. Therefore, work-family conflict may contribute to job burnout for construction professionals [12]. When job roles spill over into family roles, the time it takes for employees to get into work decreases. This leads to more work-family conflict and increased job burnout [39]. Lingard et al. [39] constructed a mediating model and justified the mediating effect of work-family conflict on the relationship between job schedule demands and burnout. Mesmer et al. [84] found that work interference with family (WIF) conflict and family interference with work (FIW) have the similar predictive effect on employees’ turnover intentions. Boyar et al. [85] and Haar [86] revealed that work-family conflict has a higher predictive power when it comes to leave intentions than family-work conflict. In addition, employees are more likely to consider leaving their jobs when work interferes with the family. Anderson et al. [60] pointed out that work interference with family (WIF) is related to work satisfaction, work stress and turnover intention, while family interference with work (FIW) is related to work stress and absentee rates. This indicates that work requirements interfering with family life will cause employee job burnout. What’s more, the immediate consequence of job burnout is the employee leaving the workforce and finding another job in order to better balance work and family demands. Therefore, the following hypothesis was developed:

**Hypothesis** **1.** *Higher levels of work-family conflict will lead to higher levels of job burnout.*

#### 3.1.2. Job Burnout and Project Performance

Cordes et al. [87] proposed that job burnout may lead to employees’ absenteeism, turnover intention and actual turnover. The most direct outcome of burnout is a reduction in work efficiency and effectivity. Burke et al. [88] pointed out that job burnout is also “contagious”. That is, an individual experiencing job burnout will also have a negative impact on his or her colleagues. The construction industry is a typical task-driven industry. To a large extent, the job burnout characteristics of construction professionals are in line with industry in general [10]. The ultimate goal of a construction enterprise is to achieve optimal project performance by controlling the project cost, improving the benefits of the project investment, completing the project within the prescribed time limit, achieving the target of the project contract and improving the comprehensive economic benefit of the enterprise [89]. The negative work behavior caused by burnout may lead to either the project not being completed on schedule or a situation of inefficiency. The depression, anxiety and other emotions caused by employees’ job burnout increase the possibility of safety accidents during the construction process, thus negatively influencing project performance. Therefore, the following hypothesis was developed:

**Hypothesis** **2.** *Higher levels of job burnout will lead to lower levels of project performance.*

#### 3.1.3. Work-Family Conflict and Project Performance

Construction professionals experience high levels of work-family conflict, due to excessive job demands and irregular and long working hours [41]. This can lead to employee dissatisfaction with both work and life, psychological stress, mental disorders, drug abuse, drinking problems, and even the willingness to risk job turnover [57,85,90,91]. Work-family conflict can lead to negative outcomes because resources are lost in the process of balancing work and family roles [81]. According to the COR theory, when resources reach minimally acceptable levels, workers stop trying their best to work to conserve personal resources and accept a decrease in performance [92]. All of these makes it difficult for employees to perform their job roles, with the ultimate result being impaired project performance. Odle-Dusseau et al. [93] confirmed the influence of work-family conflict on the job performance based on the COR theory. Frone et al. [94] and Wu et al. [15] found that work-family conflict not only reduces the quality of family life; it also leads to poor work and family performance. Goff et al. [95] pointed out that work-family conflict can significantly affect employee absenteeism, job burnout, and turnover intentions, which in turn affect project performance. In all project performance indicators, the quantitative criteria of project cost, quality and time, as well as the perception and collaboration of the participants in the whole project process, will be affected by each individual in the project team. The higher the feeling of turnover intention is, the lower the sense of accomplishment will be. Also, the more negative work mood brought about by work-family conflict is not conducive to the achievement of the above-mentioned goals. In other words, work-family conflict has a negative impact on project performance. Therefore, the following hypothesis was developed:

**Hypothesis** **3.** *Higher levels of work-family conflict will lead to lower levels of project performance.*

#### 3.1.4. The Moderating Effects of Organizational Support

Conceptually, work-family conflict is a source of role stress, and burnout is a directly-related stress response or strain response. Therefore, the relationship between work-family conflict and burnout is a stressor–strain relationship. Many occupational stress theories have argued that the extent to which people enjoy various forms of support will ease the stressor–strain relationship. For example, the COR theory suggests that people feel insecure about their ability to acquire or retain resources under stressful conditions. This feeling of insecurity can lead to emotional or physical exhaustion [96]. The stressor–strain relationship is also stronger without organizational support. However, high levels of organizational support can reduce the negative effects of work-family conflict on burnout and performance because organizational support reduce the incentive for employees to save resources among whom experiencing work-family conflict [92]. The demand-control-support (DCS) model for job stress also suggests that the most adverse health effects occur in jobs with high demand, low control, and low social support [34]. According to the DCS model, job control is not the only available resource that can be used to address work needs. Support from supervisors or colleagues can also reduce the harmful effects of stress at work and alleviate the relationship between work-family conflict and job burnout [97,98]. Therefore, this study proposed that organizational support can, to a certain extent, alleviate the job burnout caused by work-family conflict. Therefore, the following hypothesis was developed:

**Hypothesis** **4.** *Organizational support will moderate the relationship between work-family conflict and job burnout.*

### 3.2. Model Framework

**Figure 1:**
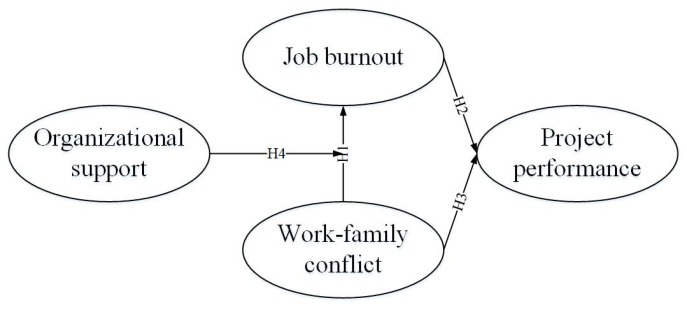
Theoretical model underlying empirical research.

Work-family conflict affects the levels of job burnout of construction professionals because of the feeling of potential or actual loss of resources. In order to minimize resource drain and protect remaining resources, they may take withdrawal behaviors [81]. Thus, resulting in a decrease in performance [92]. However, organizational support can buffer the relationship between work-family conflict and job burnout because of the resources it provides for construction professionals to achieve their work objectives, and ultimately have a positive effect on project performance. Therefore, a close relationship may exist between job burnout, work-family conflict, project performance and organizational support. Based on the developed hypotheses, and combined with the nature of construction projects, this study introduces organizational support as a moderating variable and constructs a theoretical model, as shown in Figure 1.

## 4. Variable Measurements, Questionnaire Items and Data Collection

### 4.1. Variable Measurements and Questionnaire Design

The questionnaire has three main sources of variable measured items. The first source contains the items that appear in existing literature and which have been confirmed to have a high level of reliability and validity. The second source is the items that are modified from the existing items, taking into consideration the research needs. The last source of items is set based on the characteristics of this study. In-depth interviews and exchanges with experts and scholars were conducted in construction and other relevant fields. All the variables were measured using a 7-point Likert scale (in which 1 = “strongly disagree” and 7 = “strongly agree”).

The measured variables are mainly composed of an independent variable, dependent variable and moderating variable. A total of six measurement items draw on the studies of Eisenberger [55], Hao et al. [61] and Yang et al. [2]. These items are set to measure organizational support. The job burnout scale was in accordance with reference of Lingard [39] and Yip [99]; a total of eight measurement items were set for the job burnout scale. The work-family conflict scale refers to Carlson’s [16] work-family conflict scale. A total of seven measurement items have been set up for the work-family conflict scale. The project performance scale draws on the researches of Pinto [100], Tukel et al. [101], and Joslin et al. [73]. A total of six measurement items are set for the project performance scale. The variables and their measurement items are shown in Table 1.

### 4.2. Sampling and Data Collection

This questionnaire adopts non-probabilistic sampling, which was also adopted by [102,103,104]. Respondents were selected from various construction enterprises, based on their willingness to participate. Local architectural associations realized the value of this research and with their assistance, 500 questionnaires were distributed with 438 returned. A total of 373 valid questionnaires were finally collected. This led to an effective questionnaire response rate of 74.6%. The survey locations were located in Zhejiang Province, Jiangsu Province, and Shanghai. A demographic distribution of these respondents is shown in Table 2.

## 5. Data Analysis and Results

### 5.1. Validity and Reliability

The reliability of each variable scale was tested according to its corrected-item total correlation (CITC) value and Cronbach’s α coefficient value. The Kaiser–Meyer–Olykin (KMO) test and Bartlett test were used to determine whether factor analysis could be performed. The CITC values of the measurement items of each variable scale were greater than 0.5, and all the Cronbach’s *α* values were greater than 0.8, thus indicating that the questionnaire has good reliability.

The KMO value and cumulative contribution of each variable were obtained by exploratory factor analysis. The KMO values of the four variable scales in the theoretical model were all greater than 0.6, and the Bartlett test has a Sig. <0.001, indicating that the test is suitable for factor analysis. The factor loading of each measurement item was greater than 0.6 and can be used to interpret the corresponding variable. The results are shown in Table 3.

The confirmatory factor analysis was conducted to derive the reliability of the measurement items and the reliability of factors construction. The construct reliability (CR) was used as the criterion for consistency among measurement items. The convergence validity index between the terms is the average variance extracted (AVE). Meanwhile, Chi-square static χ^2^/degrees of freedom (CMIN/DF), Root Mean Square Error of Approximation (RMSEA), Goodness-of-Fit Index (GFI), Comparative Fit Index (CFI), Adjusted Goodness-of-Fit Index (AGFI), Incremental Fit Index (IFI), and the Normed Fit Index (NFI) are used to reflect the fitness. The fitness indicators of each group of variables meet the requirements, and the standardized coefficients are all greater than 0.7. The CR value of each latent variable exceeds 0.6, indicating that the overall reliability of the measurement items and the degree of construct reliability are high. The AVE values of each latent variable are all greater than 0.5, indicating that each variable has good convergence validity. Table 4 shows the results of the confirmatory factor analysis. Therefore, the reliability and factor construct reliability of each variable met the requirements, and structural equation modeling (SEM) could be used to test the theoretical model. The χ^2^ test and different fit indexes were applied to assess the alternative models.

As shown in Table 5, the results indicated that the hypothesized model with four factors demonstrated the excellent fit. The one factor model and three-factor model demonstrated poorer fit, which were evidenced that the values of χ^2^*/df* increased, the values of CFI and TLI (Tucker-Lewis index) decreased, the values of RMSEA increased, compared with the expected four-factor model. In addition, the chi-square method was used to check for non-response bias, and the Harman one-factor test was used to test the common method bias. The results show that a significant heterogeneity exists between the variables, and the data could be seen as low common bias.

### 5.2. Hypotheses Test

Table 6 shows the results of the SEM analysis of the theoretical model. The model’s fitness index is up to standard, and the CMIN/DF value is 1.400. This not only meets the standard requirements of being below 5, but also meets the stricter requirements of being less than 3. The GFI, NFI, and IFI values are 0.930, 0.965, and 0.990, respectively, all exceed the requirement of 0.9. Meanwhile, the RMSEA value is also within the upper limit of 0.05 (at 0.037), indicating that the overall fit of the model is ideal. Thus, Hypothesis 1, 2, and 3 are all verified.

### 5.3. Moderating Effect Test

To eliminate collinearity, the independent variable and the moderating variable were first centered separately. Then, the product term of the independent variable and the manipulated variable were constructed [105]. Centralization minimizes the correlation between the product terms and the initial latent variables of the independent variable and moderating variable [106,107]. When constructing the product term, Kenny et al. [108] proposed using all possible pairs of the product term indicators of the main effect indicators (e.g., x1z1, x1z2, x2z1, x2z2). While Ping et al. [105] proposed using a single product indicator. Joreskog et al. [109] demonstrated that using a single potential variable product metric can make the data processing process more compact, with guaranteed accuracy. Therefore, the construction of single product indication was used to verify the moderating effects of organizational support. The analysis results (Table 7) showed that the index of the model was up to the standard, the fitness degree was good, and the P value corresponding to the product term was significant, so the hypothesis 4 was supported.

## 6. Discussion

### 6.1. Effects of Work-Family Conflict on Job Burnout

The results showed that work-family conflict positively affected levels of employees’ job burnout. The finding further supported the conclusions of Lingard et al. [38,39]. The specific behaviors are that construction professionals do not want to face daily work, because of their work experience. They gradually lose interests in their work, doubt the meaning of their work and even feel on the verge of collapse. The long, almost uncontrollable working hours associated with construction projects mean that construction professionals have insufficient time to participate in family activities [109]. Additionally, constant demand changes and complex tasks bring extra pressure [83], and increasing work requirements in the project’s environment may limit the ability of construction professionals to effectively complete both their work and family roles. This will ultimately trigger work-family conflict of construction professionals, eventually increasing their likelihood of experiencing job burnout. Similarly, complex and rigorous construction projects’ goals (i.e. cost, time, quality and safety) lead to heavy work demands, forcing construction professionals to spend almost all their time at work. Moreover, getting away from work does not mean the employee’s boss will not give them extra work or interruptions by phone [110]. What’s more, there will be no high productivity or significant contribution in this extra work [111]. In addition, most construction professionals are not young or single; they have spouses, children or other family members who need to be raised. The lack of time spent with family members can exacerbate work-family conflict and in turn increase the likelihood of job burnout.

### 6.2. Effects of Job Burnout on Project Performance

The results showed job burnout has a negative effect on project performance. This is consistent with the views of Maslach et al. [112] and Leung et al. [8,9]. Construction projects typically involve multiple stakeholders, and interactions amongst stakeholders directly influence project performance [113]. Employees in construction companies need to have good interpersonal skills, so that they can work well together. This is one way to ensure that the construction projects they are working on can be smoothly carried out and completed on schedule and budget. However, job burnout not only affects an individual’s daily life at home, but also causes disrespect, distrust and a dislike of colleagues [114,115]. Therefore, job burnout will affect the relationships between employees, supervisors in construction enterprises and other stakeholders in construction projects. It directly affects the construction schedule, cost, and customer’s satisfaction. Project performance is closely related to construction professionals’ morale and their perception of affiliation [8]. Construction professionals experiencing burnout may have an extreme feeling of low personal accomplishment, which in turn may lead to low levels of job satisfaction [116]. Obviously, construction professionals with a sense of low job satisfaction are unlikely to have high morale or any sense of belonging to the organization. As such, they are likely to display a series of withdrawal behaviors, such as being late for work, absenteeism, and even quitting the company, all of which will ultimately have a negative impact on project performance.

### 6.3. Effects of Work-Family Conflict on Project Performance

The results also showed work-family conflict negatively affected project performance. This was consistent with the conclusions proposed by Frone et al. [94] and Wu et al. [15]. Due to construction professionals’ feature of long working hours and complex tasks, professionals do not have enough time to spend with their families. The work-family conflict in the time dimension has a significant negative impact on the professional commitment of Chinese project employees [117], and this reduction in professional commitment leads to a reduction in project performance [118]. This finding is inconsistent with the conclusion of Allen et al. [56], who proposed that no significant relationship existed between work-family conflict and project performance. The potential explanation for this contradiction is that, with socio-economic development and career-oriented change, the pursuit of construction professionals has also shifted from simply seeking higher incomes and other economic returns to considering high-quality professional welfare, and high-quality professional welfare is significantly associated with job satisfaction. Work-family conflict leads to more psychological pressure on professionals, lowering their sense of work satisfaction and thus increasing their tendency to turnover jobs. China’s current rapid economic development provides individuals with more job opportunities and higher rotation prospects than ever before. Therefore, many construction company professionals simply quit, thus resulting in a negative effect on project performance.

### 6.4. The Moderating Effects of Organizational Support

The results showed that the degree of organizational support felt by construction professionals played a moderating role in their work-family conflict and job burnout. This finding proved to some extent that organizational support, as proposed by Yang et al. [10] and Lingard et al. [25], can alleviate the burnout of construction professionals. This finding was inconsistent with the study of Lizano et al. [119]. Perceived emotional support can provide psychological assistance; perceived instrumental support focuses on providing actual support or assistance to employees [120]. High levels of organizational support creates feelings of trust, organizational identification, and long-term commitment [121]. Organizational support also provides resources that enable professionals to accomplish work objectives. Therefore, key outcomes of organizational support include lower exit behaviors and higher job performance [122]. China has a collectivist culture, hierarchical structure and paternalistic leadership. Meanwhile, there is lack of effective supervision mechanism under the existing system, and there is a high degree of power distance. Therefore, construction professionals are sensitive to organizational concerns, and organizational support is likely to be expressed as paternalistic care. The emotional support and instrumental support felt by professionals may be important in the project context, because they want more care and guidance [104,123]. Organizational support played a role in moderating the relationship between work-family conflict and job burnout, due to their desire for more attention and supportive supervision. Hence, regular briefing meetings are advised to be held to review job progress and difficulties, with the aim to provide the construction professionals with their needed help. Some work-family balance strategies and policies such as the parent-related leave and service for their family members and enough time for construction professionals to stay with family members before the next project should also be considered. In addition, construction companies are also suggested to restrict overtime in order to ensure a balance between work and personal time for construction professionals. If they have to work overtime, proper compensation should be paid. Because the rewards provided in the form of bonuses, holidays, and promotions can make them feel worthwhile for overtime.

## 7. Conclusions and Future Work

### 7.1. Conclusions

With the development of Chinese construction industry, construction projects increasingly become with huge investment, large scale and complex tasks. This leads to long working hours, high working stress and work-family conflict, which in turn occurs job burnout. This study investigated the relationship between job burnout, work-family conflict and project performance for construction professionals in China. An empirical study is also conducted through structural equation modeling. The results showed that high levels of work-family conflict lead to high levels of job burnout, while organizational support played a role in moderating the relationship between work-family conflict and job burnout. High levels of job burnout and work-family conflict lead to low levels of project performance. If the construction companies could provide more humanistic care to their employees and provide more organizational support, they might reduce the burnout caused by work-family conflict to a certain extent, and thus improve project performance.

### 7.2. Practical Implications

The developed theoretical model has practical significance for construction enterprises, one element of which is that focusing on work-family conflict and job burnout will benefit construction enterprises and their employees. Due to long working hours, burdensome tasks and the complex interpersonal relationship, construction professionals have always experienced a high level of work-family conflict. Work-family conflict can affect an professionals’ passion and enthusiasm for work, which in turn affects job burnout levels and project performance. How to help construction professionals deal with work-family conflict and job burnout has become a critical issue that construction enterprises should concern. This study suggested that construction enterprises could provide training for construction professionals that would help solve work-family conflict. For a long time, studies have hardly revealed any adverse effects of work-family conflict on project performance. In this study, it was found that work-family conflict has both direct and indirect effects on the performance for construction professionals. Construction enterprises should not and cannot ignore the consequences of work-family conflict and job burnout, just to improve organizational efficiency and effectivity. Therefore, construction enterprises should provide a satisfactory working environment and appropriate work plan, so that construction professionals can have enough time to spend with their family members, thus reducing the level of work-family conflict. However, if they have to work overtime, appropriate rewards should be paid. Strategies and policies such as parent-related leave and service for construction professionals’ family members are suggested for construction companies to alleviate their employees’ work-family conflict. Meanwhile, the empirical results also showed that organizational support could alleviate the relationship between work-family conflict and job burnout, thereby reducing the impact of work-family conflict on project performance. Therefore, construction enterprises should establish a humanized management and communication atmosphere and culture, and formulate enterprise’s regulations and rules conducive to achieving work-family balance for employees. For example, the organization should ensure that the construction professionals have sufficient time to stay with family before assigning them to the next project. These actions would reduce the level of job burnout experienced by employees of construction enterprises caused by work-family conflict, ultimately improving project performance. In addition, frequent communication between leaders and employees is important, because two-way communication helps organizations to better understand employee confusion and then to offer support and help.

### 7.3. Limitations and Future Work

Studies on job burnout have been conducted over several decades. However, the study of job burnout for professionals in the construction industry is still lacking to an extreme degree. Furthermore, few studies investigated the relationship between job burnout, work-family conflict and project performance. This study addressed this gap by introducing organizational support as a moderating variable to develop and validate a theoretical model. The research results not only verify the existing research results, but also reveal some significant findings. However, there are still some limitations in this study. First, organizational support buffers the effects of work-family conflict on job burnout for construction professionals, but a finding suggests that more potential intermediaries may need to be investigated. For example, support from colleagues or family members may have a moderating effect on work-family conflict. Secondly, as this study was limited to the number of research samples and by restricted energy and ability, there is no further subdivision of the work-family conflict scale. This is an area upon which future research can be addressed.

## Figures and Tables

**Table 1:** Measurements for job burnout, WFC, organizational support and project performance.

Variables	Measurement Items
Job burnout	JB-1: Work makes me feel physically and mentally exhausted.
	JB-2: I feel used up after work.
	JB-3: I feel tired when I wake up in the morning and have to face the day’s work.
	JB-4: Working all day is really stressful for me.
	JB-5: Work makes me feel like I’m going to collapse.
	JB-6: I have become less and less interested in my work since I started this job.
	JB-7: I am not as enthusiastic about my work and colleagues as I used to be.
	JB-8: I doubt the meaning of my work.
Work-family conflict	WFC-1: My job requirements have affected my commitment to family responsibilities, e.g., not being able to share housework equally with my partner, or participating in family activities.
	WFC-2: Because of my family, I can’t participate in activities that will help me improve my career.
	WFC-3: Because of the pressure I feel at work, I still have no way to do what I want when I get home.
	WFC-4: The stress and anxiety felt in my family can affect my performance at work.
	WFC-5: I don’t have the interest required to participate in family activities after work.
	WFC-6: The way I handle housework is not applicable to my work.
Organizational support	OS-1: The enterprise attaches great importance to my work goals and values.
	OS-2: I can get help from my enterprise when I have trouble.
	OS-3: The enterprise values my opinion or suggestion.
	OS-4: The enterprise will allow me to have flexible working hours if I guarantee the completion of my work tasks.
	OS-5: The time off I have at work or outside of work is enough for me to deal with my family chores.
	OS-6: The enterprise really cares about my happiness.
Project performance	PP-1: The project I am participating in is or will be completed on schedule.
	PP-2: The current project is or will be completed on budget.
	PP-3: The project’s results or deliverables meet expected goals.
	PP-4: If a problem arises, a friendly solution is generally found.
	PP-5: Partners are satisfied with the process of the project’s completion.
	PP-6: The current partners are willing to cooperate with each other in the future.

**Table 2:** Demographics of respondents.

	Category	Frequency	Percentage (%)
Province/City	Zhejiang Province	124	33.24
	Jiangsu Province	159	42.63
	Shanghai	90	24.13
Education background	Junior college and below	158	42.36
	Bachelor	184	49.33
	Master	24	6.43
	Doctor	7	1.88
Designation	Project manager	42	11.26
	Department manager	45	12.06
	Project engineer	86	23.06
	Professional manager	69	18.50
	Site builder	105	28.15
	Others	26	6.97
Work experience	<5 years	99	26.54
	6–10 years	123	32.97
	11–15 years	107	28.69
	16–20 years	28	7.51
	>20 years	16	4.29

**Table 3:** Results of validity and reliability.

Variables	Measurements	Factor Loading	Cronbach’s α	KMO	Variance Explained
Job burnout	JB-1	0.890	0.964	0.963	79.7%
	JB-2	0.896			
	JB-3	0.882			
	JB-4	0.886			
	JB-5	0.895			
	JB-6	0.897			
	JB-7	0.890			
	JB-8	0.904			
Work-family conflict	WFC-1	0.872	0.942	0.923	77.8%
	WFC-2	0.885			
	WFC-3	0.900			
	WFC-4	0.887			
	WFC-5	0.840			
	WFC-6	0.904			
Organizational support	OS-1	0.888	0.946	0.937	78.8%
	OS-2	0.893			
	OS-3	0.889			
	OS-4	0.888			
	OS-5	0.889			
	OS-6	0.881			
Project performance	PP-1	0.904	0.956	0.937	82.0%
	PP-2	0.913			
	PP-3	0.908			
	PP-4	0.903			
	PP-5	0.906			
	PP-6	0.898			

**Table 4:** Results of confirmatory factor analysis.

Variables	Measurements	Standardized Coefficient	CR	AVE	Fitness Indicators
Job burnout	JB-1	0.87	0.96	0.77	CMIN/DF = 1.349
	JB-2	0.88			RMSEA = 0.034
	JB-3	0.86			GFI = 0.976
	JB-4	0.87			AGFI = 0.956
	JB-5	0.88			NFI = 0.989
	JB-6	0.88			IFI = 0.997
	JB-7	0.87			
	JB-8	0.89			
Work-family conflict	WFC-1	0.84	0.94	0.73	CMIN/DF = 3.634
	WFC-2	0.86			RMSEA = 0.095
	WFC-3	0.89			GFI = 0.964
	WFC-4	0.86			AGFI = 0.915
	WFC-5	0.79			NFI = 0.979
	WFC-6	0.89			IFI = 0.985
Organizational support	OS-1	0.86	0.95	0.74	CMIN/DF = 1.744
	OS-2	0.87			RMSEA = 0.045
	OS-3	0.87			GFI = 0.993
	OS-4	0.86			AGFI = 0.983
	OS-5	0.86			NFI = 0.996
	OS-6	0.85			IFI = 0.901
Project performance	PP-1	0.88	0.96	0.78	CMIN/DF = 1.926
	PP-2	0.90			RMSEA = 0.056
	PP-3	0.89			GFI = 0.981
	PP-4	0.88			AGFI = 0.955
	PP-5	0.89			NFI = 0.990
	PP-6	0.87			IFI = 0.995

**Table 5:** The comparative results of alternative models.

Models Used to Discriminate the Measures	χ^2^/*df*	CFI	TLI	RMSEA	GFI
One-factor model: all the factors merged	25.08	0.073	0.067	0.290	0.134
Three-factor model: JB+WFC, OS, PP	2.217	0.956	0.954	0.064	0.810
Four-factor model: JB, WFC, OS, PP	1.273	0.991	0.990	0.030	0.913

Notes: JB means job burnout; WFC means work-family conflict, OS means organizational support; PP means project performance.

**Table 6:** The SEM results of theoretical model.

Relationship between Variables	Standardized Coefficients	Hypotheses Supported
Work-family conflict → job burnout Job burnout → project performance Work-family conflict → project performance	0.89 *	H1: supported
	−0.49 *	H2: supported
	−0.41 *	H3: supported
Fit indices	CMIN/DF = 1.400; RMSEA = 0.037; GFI = 0.930; AGFI = 0.912; NFI = 0.965; IFI = 0.990	

Notes: * indicates *p* < 0.05.

**Table 7:** The results of moderating effect analysis.

Relationship between Variables	Standardized Coefficients	P
Work-family conflict → job burnout	0.404	***
Organizational support → job burnout	−0.505	***
Work-family conflict × Organizational support → job burnout	−0.024	***
Fit indices	CMIN/DF = 1.507; RMSEA = 0.043; GFI = 0.919; AGFI = 0.900; NFI = 0.957; IFI = 0.985	

Notes: *** indicates *p* < 0.001.
